# Higher than expected and significantly increasing incidence of upper tract urothelial carcinoma. A population based study

**DOI:** 10.1007/s00345-020-03576-3

**Published:** 2021-01-09

**Authors:** Bjarte Almås, Ole J. Halvorsen, Tom Børge Johannesen, Christian Beisland

**Affiliations:** 1 Department of Urology, Haukeland University Hospital, 5021 Bergen, Norway; 2 Cancer Registry of Norway, Ullernchausseen 64, 0379 Oslo, Norway; 3 Department of Pathology, Haukeland University Hospital, 5021 Bergen, Norway; 4 Department of Clinical Medicine, University of Bergen, Bergen, Norway; 5 Centre for Cancer Biomarkers CCBIO, Department of Clinical Medicine, Section for Pathology, University of Bergen, Bergen, Norway

**Keywords:** Upper tract urothelial carcinoma, Epidemiology, Incidence, Registry data, Population based study

## Abstract

**Purpose:**

To register all cases of urothelial cancer and renal cell carcinoma (RCC) in Norway during 1999–2018 to obtain the contemporary incidence of UTUC and UTUC incidence relative to other urothelial cancers and RCC. Further to analyse possible changes over time regarding UTUC incidence, UTUC patient characteristics, tumour characteristics and survival.

**Methods:**

3502 cases registered with ICD code C65 and C66 during 1999–2018 at the Norwegian cancer registry were entered into a database. After a selection process 3096 cases were included in the study. The crude incidences of UTUC were calculated for each year adjusting for the corresponding population data. Age-standardized rates adjusting to the European standard population (2013) were calculated. Comparisons were made with other cases of urothelial cancer and RCC. For changes over time, the material was split into 5-year periods. Regression analysis was used to calculate yearly changes and for assessing statistical significance. Survival outcomes were calculated using the Kaplan–Meier method.

**Results:**

The overall age-standardized incidence rate was 3.88, increasing from 3.21 to 4.70 from first to last 5-year periods. The increase affected all ages except those < 60 years of age, and were observed regardless of gender or anatomical location. UTUC constituted 11.8% of all urothelial cancers, increasing from 9.9 to 12.8%. Mean patient age at diagnosis increased from 71.5 to 73.4 years. The 5-years Cancer-specific survival improved from 57.4 to 65.4%.

**Conclusion:**

The incidence of UTUC was higher than expected and increasing. Patient age at diagnosis was increasing.

**Supplementary Information:**

The online version contains supplementary material available at 10.1007/s00345-020-03576-3.

## Introduction

Compared to urothelial cancer of the bladder (BC), upper tract urothelial carcinoma (UTUC) is relatively uncommon. The incidence is typically referred to be 1–2:100.000 per year or 5–10% of all urothelial carcinomas. Urothelial cancer in the kidney pelvis has been referred to constitute 7% of all renal tumours. As a source of these numbers, the yearly publication from the American Cancer Society is often quoted [1]. In the yearly publication from the Norwegian national database at the Cancer Registry of Norway (NCR) all new cancer cases of UTUC are merged with cases of the much more common BC and cancer of the urethra [2]. Specific contemporary data regarding the incidence of UTUC and changes over time is limited. Some authors have reported an increasing incidence of UTUC [3, 4], while others have reported a stable incidence, or even a decline [5, 6]. To our knowledge, currently published papers on this topic do not include patient cohorts after 2011. Basic epidemiological knowledge is essential in the planning of diagnostic evaluations, treatment and research of a particular disease. We, therefore, decided to gather and analyse available data regarding UTUC from the NCR in Norway during 1999–2018. We formulated the following aims for the study.

### Primary objective

To register all cases of UTUC together with all other cases of urothelial cancer and renal cell carcinoma (RCC) in Norway during 1999–2018 to obtain the contemporary incidence of UTUC in Norway together with UTUC incidence relative to other urothelial cancers and RCC.

### Secondary objective

To look for and analyse possible changes over time regarding UTUC incidence, patient and tumour characteristics and survival outcomes.

## Material and method

All patients classified with the International Classification of Diseases tenth revision [7] (ICD-10) diagnosis code C65 (cancer in the kidney pelvis) and C66 (cancer in the ureter) registered during 1999–2018 were extracted from the main database at NCR. A dataset of 3502 cases was obtained. For comparison, a similar extraction was made for renal cell carcinoma (RCC, C64, *n* = 14,500), BC (C67, *n* = 27,427 and Urethral cancer (C68, *n* = 440). The database included information about patient sex, age at diagnosis, date of birth, histopathological data, clinical data (cancer report, death report including the cause of death etc.), treatment and current status (deceased or alive). The data from the NCR include nodal status and metastasis at diagnosis if present, but complete data on pathological or clinical tumour stage is not available. As a substitute, the tumours are coded as invasive (pT2 +) or non-invasive (pTa/T1). This classification is available for pure urothelial carcinomas only (see Table 1). The inclusion/exclusion process is illustrated in supplementary Fig. 1. In the case of diagnostic uncertainty, the cases were examined manually together with NCR personnel to clarify the basis of the diagnosis code and consider if the cases could be included or not. Of 1026 uncertain UTUC cases, 305 were excluded, typically where the diagnosis code was based on atypical cells by cytology or biopsy, when the tumour was coded wrong and was benign (i.e. benign papilloma) or when there was doubt whether the cells were benign or malignant. In 29 cases, the diagnosis code was based on very sparse information, i.e. only a death report or a clinical report based on autopsy or clinical examination, and these were excluded. In addition, 72 cases of pure non-urothelial cancers (i.e. squamous cell carcinoma or adenocarcinoma) were excluded. All cases of pure urothelial carcinoma and cases of urothelial carcinoma with divergent differentiation were included. A comprehensive list of included and excluded cases is shown in Table 1. In all, 3096 cases in 2818 patients were included in the final analysis. More than one case of UTUC on the same patient was uncommon and only registered if data suggested a truly new tumour, i.e. considerable time between cases or a new tumour on the opposite side. All analyses were performed according to number of cases, not number of patients. Of all 3096 cases, 2969 (95.9%) were verified with histopathological examination of a surgical specimen (*n* = 2327, 75.2%), biopsy (*n* = 576, 18.6%) or cytology (*n* = 66, 2.1%). In the remaining 127 (4.1%), the basis was a clinical report using radiological examination, endoscopic procedure or radiation therapy data as sources for the diagnosis codes. Similar inclusion and exclusion processes were performed regarding BC, urethral cancer and RCC resulting in 24,467 included cases of BC, 13,619 with RCC and 287 with urethral cancer.

**Table 1:** Description of included and excluded cases in the study

Tumour characteristics	*n*	%^a^
All cases	3502	100
Included	3096	88.4
Pure urothelial carcinoma	2856	81.6
Urothelial carcinoma with divergent differentiation	45	1.3
Carcinoma in situ	68	1.9
No histopathological verification	127	3.6
Excluded	406	11.6
Other malignant tumour	72	2.1
Squamous cell carcinoma	27	0.8
Adenocarcinoma	26	0.7
Sarcoma	9	0.3
Lymphoma	5	0.1
Neuroendocrine tumour	4	0.1
Other	1	< 0.1
Benign tumour/uncertain	305	8.8
Urothelial Atypia/dysplasia etc	288	8.2
Benign tumour	17	0.5
Limited data available	29	0.8
Death certificate only	11	0.3
Autopsy only	8	0.2
Clinical examination only	10	0.3

^a^Percentages given as % of both included and excluded cases and might differ from % in manuscript

For incidence rates, crude rates were calculated using population data in Norway corresponding to each year from 1999 to 2018. To adjust for demographic differences between the Norwegian and other populations, age-standardized rates (ASR) according to the European standard population published in 2013 were calculated [8]. ASRs adjusted to other available standard populations were also calculated for comparison (see supplementary Table 1 and 2).

### Statistical analysis

For the purpose of analysing changes over time, the material was split into 5-year periods (1999–2003, 2004–2008, 2009–2013 and 2014–2018). The relative proportion of UTUC cases compared to all urothelial cancer cases and pelvic urothelial tumour cases compared to RCC cases were calculated for each 5-year periods. Analyses regarding potential changes in patient age, gender distribution, location of the tumour and tumour features were performed in the same manner. For further analyses of changes over time, the estimated average percentage changes (EAPC) for incidence rates were calculated and linear regression analyses were used to calculate yearly changes and for assessing statistical significance. Survival analyses included both overall survival (OS) and cancer-specific survival (CSS) and were performed using the Kaplan–Meier method. Categorical data were analysed using the Chi-square method. Data were analysed using IBM® SPSS® Statistics v. 26. *P *values less than 0.05 were considered statistically significant.

## Results

**Fig. 1:**
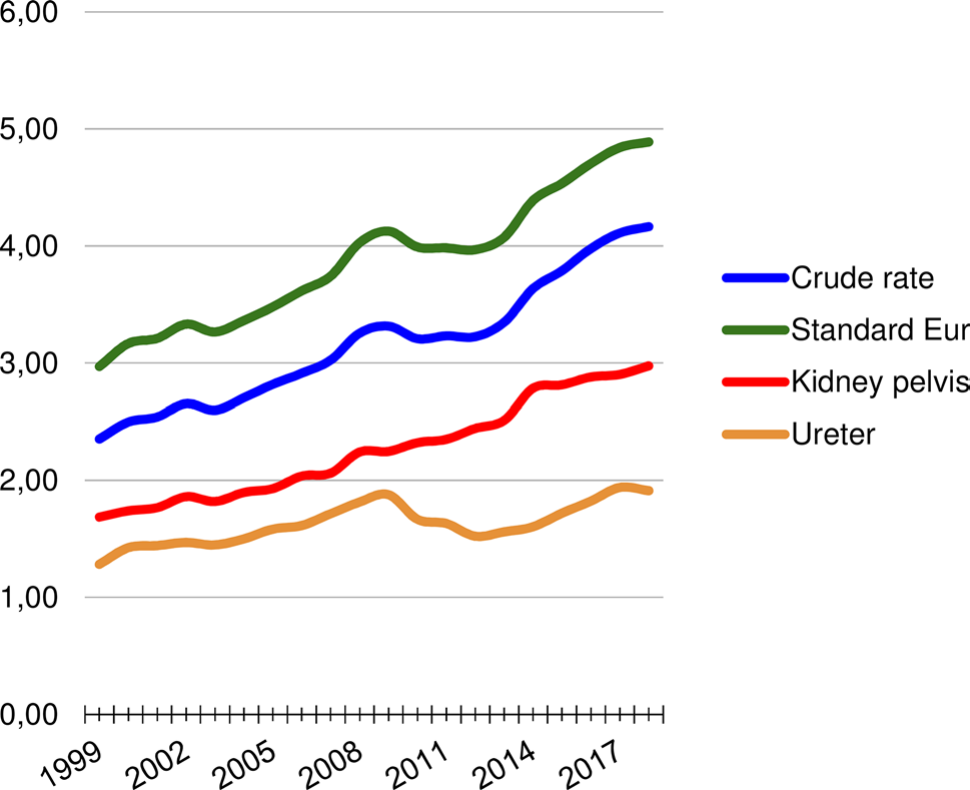
Demonstrates the 5-years average UTUC incidence per 100.000 and changes over time. Illustrates the crude rates (blue) and the age-standardized incidence rates adjusted to the European standard population, 2013 version, green). Includes the incidence rates of UTUC in the kidney pelvis (red) and the ureter (orange)

The developments in crude rates and ASRs of UTUC during the study period are illustrated in Fig. 1. Specific ASR regarding UTUC in the kidney pelvis and the ureter are also included in the figure. The crude incidence of UTUC in the whole time period was 3.17:100.000, increasing from 2.54 to 3.98 from the first to last 5 year periods. The estimated annual increase was 0.09 (CI 0.07–0.12), *p* < 0.001) resulting in an EAPC of 3%. The ASR adjusted to the European standard population was 3.88 for the whole period, increasing from 3.21 to 4.70. The increase per year was 0.10 (CI 0.06–0.13, *p* < 0.001) with an EAPC of 2.5%. The ASR of UTUC in the kidney pelvis increased from 1.77 to 2.88 from first period to last, *p* < 0.001. For ureteral tumours the increase was from 1.44 to 1.82 during the same period, *p* < 0.001. The proportion of tumours in the renal pelvis compared to all UTUC increased non-significantly from 55.6 to 61.2%, *p* = 0.06. The ASRs adjusted to other standard populations are presented in Supplementary Table 1 and 2.

Analyses showed that UTUC incidence increased in all age-spans above 60 years age, see supplementary Fig. 2. There was no increase over time in new yearly UTUC cases among patients under the age of 60, but comparing first 5-year periods with the last the increase was apparent and significant for each decade from 60 to 69 years (131–254 cases, 94% increase) 70–79 years (243–399 cases, 64% increase) and 80 + years (121–303 cases, 150% increase), all *p* < 0.001.

Patient demographics, tumour features and developments over time are shown in Table 2. The table also includes comparisons between UTUC and other urothelial cancers and RCC.

**Table 2:** Changes over time regarding patient demographics, tumour features and new cases of upper tract urothelial carcinoma compared to other urothelial cancers and renal cell carcinoma

Variable	All	1999–2003	2004–2008	2009–2013	2014–2018	%^a^	*p*^b^
Mean age (years)	72.8	71.8	72.0	72.6	73.9		< 0.001
Gender %							
Female	37	41.1	32.3	39.3	36.1		0.3
Male	63	58.9	67.7	60.7	63.9		
Location							
Kidney pelvis	58.5	55.6	56.1	59.1	61.2		0.06
Ureter	41.5	44.4	43.9	40.9	38.8		
Tumour stage %							
Invasive (T2–T4)	46.9	50.0	47.7	50.7	41.7		0.07^c^
Non-invasive (Ta–T1)	41.4	42.5	42.7	39.1	41.8		
Invasiveness not assessable (Tx)	11.7	7.5	9.5	10.1	16.5		< 0.001
Regional node metastases	5.2	4.7	5.4	6.6	4.2		0.9
Distant metastases	9.6	9.9	10.4	9.8	8.8		0.5
Upper tract urothelial carcinoma (*n*)	3096	574	681	800	1041	81	< 0.001
Bladder cancer	24,467	5251	5910	6181	7125	36	< 0.001
Urethral cancer	287	61	74	54	98	61	0.2
Total Urothelial cancer	27,850	5886	6665	7035	8264	40	< 0.001
% Upper tract urothelial carcinoma	11.1	9.8	10.2	11.4	12.6	29	< 0.001
Renal tumours (*n*)							
Urothelial carcinoma kidney pelvis	1811	319	382	473	637	100	< 0.001
Renal cell carcinoma	13,619	2501	3103	3711	4304	72	< 0.001
Total	15,430	2820	3485	4184	4941	75	< 0.001
%Upper tract urothelial carcinoma	11.7	11.3	11.0	11.3	12.9	14	0.04

^a^Increase in percent from first to last 5-year periods

^b^*p* values based on regression analyses assessing yearly changes

^c^Chi-square comparing invasive with non-invasive

Mean (median, IQR) age of all UTUC patients during the whole period was 72.8 (73.8, 65.8–79.8) years. Patient mean age at diagnosis increased from 71.8 to 73.9 from the first to last 5 year periods, *p* < 0.001. No gender-specific changes over time were observed.

No statistically significant stage migration over time was observed. The proportion of invasive tumours decreased non-significantly from 50.0 to 41.7% compared to non-invasive tumours (*p* = 0.07). Invasive tumours were equally frequent irrespective of gender or age. Similarly, analyses were performed regarding regional node or distant metastases, but no differences over time were observed for the entire cohort or stratified by age or gender. The proportion of cases where invasiveness was not assessable increased over time, corresponding to an increase in cases verified by biopsy only, and a decrease in radical surgery.

During the whole study period, 75.2% of the patients were treated with radical surgery. The absolute number of patients treated with radical surgery increased by 55.5% during the study period, but since number of cases increased by 81.4%, there was a net decline in the *proportion* of patients treated with radical surgery over time from 82.6 to 70.8% (*p* < 0.001). The proportion of patients with a biopsy verified diagnosis without following radical surgery increased correspondingly from 10.8 to 23.7% in the same period.

Regarding the oldest patients (> 80 years of age) fewer patients (59.3%) were treated with radical surgery, decreasing from 64.5 to 55.6% in the study period. More of these patients were diagnosed with biopsy without following radical treatment, increasing from 18.2 to 31.2% in the study period. Among these oldest patients, it was also more common that the diagnosis was not verified with a histopathological specimen, (16.0% vs 2.8% for patients < 80 years age), stable during the study period.

**Fig. 2:**
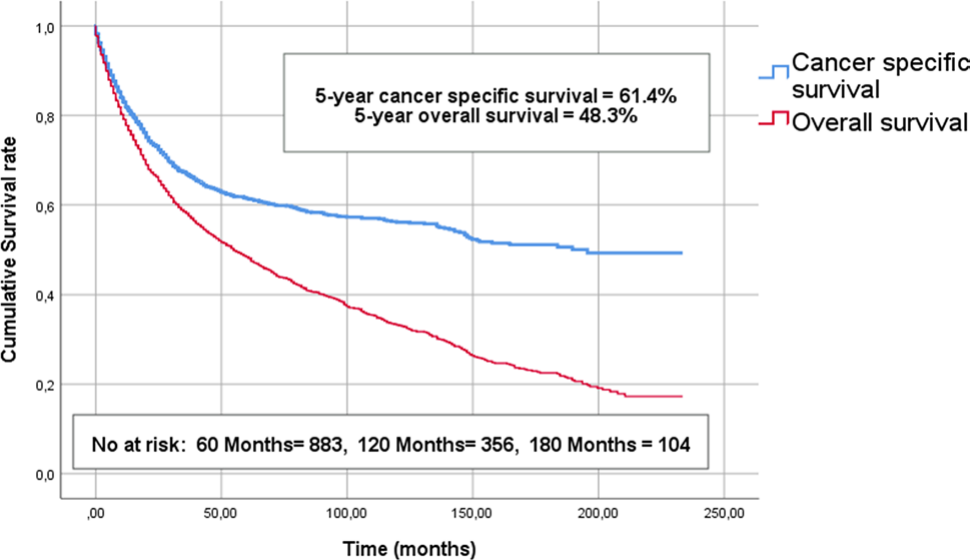
Shows the estimated overall- and cancer-specific survival curves of the entire cohort using the Kaplan–Meier method. The 5, 10 and 15-years OS were 48.3%, 33.2% and 22.5%, respectively. The 5, 10 and 15-years CSS were 61.4%, 56.1% and 51.1%, respectively

The 5, 10 and 15-years OS were 48.3%, 33.2% and 22.5%, respectively. The 5, 10 and 15-years CSS were 61.4%, 56.1% and 51.1%, respectively (Fig. 2). All the following survival data are given as 5-years CSS. Patients treated with radical surgery had significantly higher survival compared to patients not treated with radical surgery (67.2% vs 41.6%, *p* < 0.001), respectively. The patients with non-invasive tumours had higher survival compared to patients with invasive UTUC (79.4% vs 49.8%, *p* < 0.001). Survival deteriorated with increasing age, patients < 70 years 68.1%, 70–80 age 63.5% and > 80 years of age 46.7%, respectively, *p* < 0.001. No differences in survival stratified by gender or tumour location were detected.

Both OS and CSS improved over time, comparing the last decade with the first (5-year OS 44.0% vs 53.2%, *p* ≤ 0.001 and 5-year CSS 57.4% vs 65.4%, *p* ≤ 0.001), respectively. This improvement over time was present for all sub-groups irrespective of age, gender, type of treatment and tumour location.

## Discussion

If the commonly quoted incidence rate of 1–2:100.000 from the American Cancer society serves as a reference, the ASR of 4.7:100.000 in the present study was higher than expected, and to our knowledge the highest incidence rate published based on a population outside endemic areas. There was an estimated annual percentage change in the incidence rate of 2.5%, corresponding to an 81% increase in new cases comparing first with last 5-year periods.

The reason for the demonstrated increase is not clear. One possible explanation could be that symptoms i.e. haematuria are more vigorously examined in older patients now than before. An improved access to high-quality computed tomography and better equipment for flexible ureteroscopy could add to this effect. This could lead to a higher detection rate of UTUC and increased age at diagnosis. Indeed, we have seen a considerable increase in biopsy verified cases without following radical surgeries. This increase is even more evident among patients > 80 years of age, the same age group that showed the greatest increase in new yearly cases. These data indicate that at least some of the demonstrated increase in incidence could be due to increased diagnostics, especially among the oldest patients.

The decrease in radical treatments could be caused by an increased use of endoscopic treatment. Unfortunately, the data from the NCR does not include data on endoscopic treatment. However, an increasing number of publications with relatively larger cohorts on the use of endoscopic treatment could indicate an increased use of this treatment modality [9, 10]. Another reason could be that observation was chosen over radical surgery due to high age, poor performance status or favourable tumour characteristics. Data on performance status are not available in the present dataset, but a considerable and increasing proportion of patients were at a high age where larger surgeries might not be recommended.

In spite of these possible reasons for the described increase, it seems likely that there is a true increase in UTUC in Norway for the last 20 years, and not just an increased detection rate. The known risk factors for UTUC include smoking [11, 12], excessive alcohol consumption and exposure to aristolochic acid [13]. The dataset obtained from the NCR does not contain information about smoking, alcohol use or exposure to possible carcinogens. An evaluation about the potential effect of changing exposure to known risk factors is for this reason not possible without obtaining further data and was beyond the scope of this paper. Further studies to clarify the reasons for the described increase are needed.

In the present cohort, we found an all-cohort 5-years CSS and OS of 61.4% and 48.3%, respectively. Other population-based publications on UTUC which include survival data have demonstrated similar survival outcomes. Raman et al. presented a 5-years OS of ~ 50% in their cohort [3], while Eylert et al. reported a falling 5-years relative survival rate from 60 to 48% during their study period [14]. Woodford et al. reported a 5-years overall survival rate of 32% [5]. Compared to these more historic cohorts, the present study demonstrated comparable or favourable survival outcomes. A moderate improvement in survival over time was observed. The reason for this improvement is unclear. Increased use of adjuvant therapies for UTUC including both perioperative chemotherapy [15, 16] and the introduction of immunotherapies [17] could possibly explain some of the demonstrated improved survival in the present cohort. Unfortunately, the data at NCR is very limited regarding the use of adjuvant treatment, and no firm conclusions can be drawn.

As UTUC is a potentially lethal disease if left untreated, one would expect that earlier detection and treatment could result in improved survival. In the present cohort, we found an increased use of biopsies without following radical treatment. As stated earlier, the present data set is not complete regarding tumour stage, but a non-significant decline in the proportion of invasive tumours was observed. It is possible that more cases are detected at an earlier stage presently compared to previously, resulting in improved survival.

Our findings could have several possible implications. One implication could be an increased focus on UTUC, simply because more patients than expected would be affected by the disease. Another implication could be enrolment into studies. There are many unanswered questions regarding the diagnostic work-up and treatment of UTUC, such as the optimal use of perioperative chemotherapy or the use of lymph node dissection at the time of RNU. A higher incidence would result in quicker enrolment into much needed studies on the topic, and make studies with adequate patient numbers more feasible to conduct.

### Strengths and weaknesses

The present publication is based on national data from Norway, analysing 3096 UTUC cases during 20 years, a sufficient number of cases to make reliable conclusions about a relatively rare disease. The NCR is nationwide and has since 1953 kept a complete registry of all new cases of malignant neoplasms. It has documented a high degree of data quality including key aspects such as completeness, comparability and validity [18]. The data material was quality assured, based on clinical and pathology reports, and statistical advice was sought to make sure the methods used for incidence measurements, population adjustment and changes over time were performed in the correct way.

This study is not without limitations. One weakness of the study is that the analyses were based on registry data partly based on clinical reports made from a wide range of clinicians, with an inherent risk of coding errors. More specifically the dataset is limited by a lack of accurate data regarding tumour stage and specific data on treatment i.e. the use of endoscopic treatment. The data also has limitations regarding the registration of CIS, prior bladder cancer, race and the use of adjuvant treatments. As the present study is a population-based registry study with the described limitations, the ability to draw firm conclusions about the causality concerning our findings is limited. Further studies to explore further possible reasons for the increased incidence, changing demographics and improved survival are warranted.

## Conclusion

The incidence of UTUC was higher than previously reported, and increasing. UTUC incidence in Norway during 2014–2018 was 4.7:100.000. UTUC currently constitutes close to 13% of all urothelial cancers, and urothelial cancers of the renal pelvis currently constitute close to 13% of all malignant renal tumours. The increase was not accompanied by stage migration, but survival moderately improved. The patients are older at the time of diagnosis currently compared to earlier, but no other changes in patient demographics were detected.

## Supplementary Information

Below is the link to the electronic supplementary material.

Supplementary file1 (DOCX 17 KB)

Supplementary file2 (DOCX 19 KB)

Supplementary file3 (TIF 350 KB)

Supplementary file4 (TIF 1495 KB)

## Data Availability

The data material used for the study can be inquired from the corresponding author if necessary.

## Code availability

Only simple coding and common statistics software was used.

## References

[1] Siegel RL, Miller KD, Jemal A (2020). Cancer statistics, 2020. Cancer J Clin.

[2] Larsen IK (2017) Cancer in Norway 2017—cancer incidence, mortality, survival and prevalence in Norway. In: edn. Oslo, Norway: Norwegian Cancer registry. pp 40–42

[3] Raman JD, Messer J, Sielatycki JA, Hollenbeak CS (2011). Incidence and survival of patients with carcinoma of the ureter and renal pelvis in the USA, 1973–2005. BJU Int.

[4] Cauberg EC, Salomons MA, Kummerlin IP (2010). Trends in epidemiology and treatment of upper urinary tract tumours in the Netherlands 1995–2005: an analysis of PALGA, the Dutch national histopathology registry. BJU Int.

[5] Woodford R, Ranasinghe W, Aw HC, Sengupta S, Persad R (2016). Trends in incidence and survival for upper tract urothelial cancer (UTUC) in the state of Victoria-Australia. BJU Int.

[6] Wihlborg A, Johansen C (2010). Incidences of kidney, pelvis, ureter, and bladder cancer in a nationwide, population-based cancer registry, Denmark, 1944–2003. Urology.

[7] ICD-10 (2004) International statistical classification of diseases and related health problems: tenth revision. World Health Organization 3376487

[8] The Human cause-of-death Database https://www.causesofdeath.org/docs/standard.pdf

[9] Scotland KB, Hubbard L, Cason D et al (2020) Long term outcomes of ureteroscopic management of upper tract urothelial carcinoma. Urol Oncol 10.1016/j.urolonc.2020.06.027 32773230

[10] Villa L, Haddad M, Capitanio U (2018). Which patients with upper tract urothelial carcinoma can be safely treated with flexible ureteroscopy with holmium: YAG laser photoablation? Long-term results from a high volume institution. J Urol.

[11] McLaughlin JK, Silverman DT, Hsing AW (1992). Cigarette smoking and cancers of the renal pelvis and ureter. Can Res.

[12] Crivelli JJ, Xylinas E, Kluth LA, Rieken M, Rink M, Shariat SF (2014). Effect of smoking on outcomes of urothelial carcinoma: a systematic review of the literature. EurUrol.

[13] Roupret M, Babjuk M, Comperat E (2018). European association of urology guidelines on upper urinary tract urothelial carcinoma: 2017 update. EurUrol.

[14] Eylert MF, Hounsome L, Verne J, Bahl A, Jefferies ER, Persad RA (2013). Prognosis is deteriorating for upper tract urothelial cancer: data for England 1985–2010. BJU Int.

[15] Seisen T, Krasnow RE, Bellmunt J (2017). Effectiveness of adjuvant chemotherapy after radical nephroureterectomy for locally advanced and/or positive regional lymph node upper tract urothelial carcinoma. J Clin Oncol.

[16] Birtle A, Johnson M, Chester J (2020). Adjuvant chemotherapy in upper tract urothelial carcinoma (the POUT trial): a phase 3, open-label, randomised controlled trial. Lancet (London, England).

[17] Rosenberg JE, Hoffman-Censits J, Powles T (2016). Atezolizumab in patients with locally advanced and metastatic urothelial carcinoma who have progressed following treatment with platinum-based chemotherapy: a single-arm, multicentre, phase 2 trial. Lancet (London, England).

[18] Larsen IK, Smastuen M, Johannesen TB (2009). Data quality at the Cancer Registry of Norway: an overview of comparability, completeness, validity and timeliness. Eur J Cancer (Oxford, England: 1990).

