# Supplementary material for: Higher than expected and significantly increasing incidence of upper tract urothelial carcinoma. A population based study
Source: World J Urol. 2021 Jan 9;39(9):3385–91. doi: 10.1007/s00345-020-03576-3 (PMC8510951; doi:10.1007/s00345-020-03576-3)
Supplement: Supplementary file 1 — Supplementary file1 (DOCX 17 KB) [file 345_2020_3576_MOESM1_ESM.docx]

| Age | European standard 2013 | European standard 1976 | Ameri can standard 2000 | World standard 1966 | Nordic standard 2000 |
| --- | --- | --- | --- | --- | --- |
| 00-04 | 0,05 | 0,08 | 0,069135 | 0,120 | 0,059 |
| 05-09 | 0,055 | 0,07 | 0,072533 | 0,100 | 0,066 |
| 10-14 | 0,055 | 0,07 | 0,073032 | 0,090 | 0,062 |
| 15-19 | 0,055 | 0,07 | 0,072169 | 0,090 | 0,058 |
| 20-24 | 0,06 | 0,07 | 0,066478 | 0,080 | 0,061 |
| 25-29 | 0,06 | 0,07 | 0,064529 | 0,080 | 0,068 |
| 30-34 | 0,065 | 0,07 | 0,071044 | 0,060 | 0,073 |
| 35-39 | 0,07 | 0,07 | 0,080762 | 0,060 | 0,073 |
| 40-44 | 0,07 | 0,07 | 0,081851 | 0,060 | 0,07 |
| 45-49 | 0,07 | 0,07 | 0,072118 | 0,060 | 0,069 |
| 50-54 | 0,07 | 0,07 | 0,062716 | 0,050 | 0,074 |
| 55-59 | 0,065 | 0,06 | 0,048454 | 0,040 | 0,061 |
| 60-64 | 0,06 | 0,05 | 0,038793 | 0,040 | 0,048 |
| 65-69 | 0,055 | 0,04 | 0,034264 | 0,030 | 0,041 |
| 70-74 | 0,05 | 0,03 | 0,031773 | 0,020 | 0,039 |
| 75-79 | 0,04 | 0,02 | 0,026999 | 0,010 | 0,035 |
| 80-84 | 0,025 | 0,01 | 0,017842 | 0,005 | 0,024 |
| 85+ | 0,025 | 0,01 | 0,015508 | 0,005 | 0,019 |
|  | 1 | 1 | 1 | 1 | 1 |

Supplementary table 1. Table showing different standard populations that can be used for calculation of age standardized incidence rates. Note the different weights especially at high ages. This causes the relatively large differences in age standardized incidence rates demonstrated in supplementary table 2.
