# Supplementary material for: Higher than expected and significantly increasing incidence of upper tract urothelial carcinoma. A population based study
Source: World J Urol. 2021 Jan 9;39(9):3385–91. doi: 10.1007/s00345-020-03576-3 (PMC8510951; doi:10.1007/s00345-020-03576-3)
Supplement: Supplementary file 2 — Supplementary file2 (DOCX 19 KB) [file 345_2020_3576_MOESM2_ESM.docx]

| Year | Crude rate | European standard 2013 | European standard 1976 | American standard 2000 | World standard 1966 | Nordic standard 2000 |
| --- | --- | --- | --- | --- | --- | --- |
| 1999 | 2,35 | 3,00 | 1,93 | 2,01 | 1,30 | 2,50 |
| 2000 | 2,47 | 3,08 | 1,94 | 2,10 | 1,30 | 2,60 |
| 2001 | 2,24 | 2,83 | 1,88 | 1,94 | 1,28 | 2,38 |
| 2002 | 2,93 | 3,76 | 2,31 | 2,51 | 1,51 | 3,12 |
| 2003 | 2,72 | 3,39 | 2,05 | 2,28 | 1,32 | 2,87 |
| 2004 | 2,92 | 3,61 | 2,22 | 2,43 | 1,47 | 3,03 |
| 2005 | 2,18 | 2,74 | 1,74 | 1,87 | 1,15 | 2,31 |
| 2006 | 2,77 | 3,32 | 2,04 | 2,40 | 1,40 | 2,89 |
| 2007 | 3,50 | 4,34 | 2,64 | 2,92 | 1,72 | 3,64 |
| 2008 | 3,19 | 4,07 | 2,51 | 2,76 | 1,65 | 3,44 |
| 2009 | 3,48 | 4,23 | 2,61 | 2,87 | 1,70 | 3,59 |
| 2010 | 3,33 | 4,17 | 2,47 | 2,79 | 1,59 | 3,49 |
| 2011 | 3,07 | 3,82 | 2,37 | 2,61 | 1,55 | 3,25 |
| 2012 | 2,97 | 3,66 | 2,13 | 2,42 | 1,36 | 3,05 |
| 2013 | 3,31 | 4,04 | 2,47 | 2,75 | 1,62 | 3,43 |
| 2014 | 3,45 | 4,16 | 2,47 | 2,83 | 1,60 | 3,53 |
| 2015 | 3,95 | 4,70 | 2,75 | 3,17 | 1,77 | 3,99 |
| 2016 | 4,51 | 5,39 | 3,06 | 3,59 | 1,93 | 4,52 |
| 2017 | 3,73 | 4,38 | 2,53 | 2,93 | 1,59 | 3,68 |
| 2018 | 4,25 | 4,89 | 2,85 | 3,25 | 1,82 | 4,08 |
|  |  |  |  |  |  |  |
| 1999-2003 | 2,54 | 3,21 | 2,02 | 2,17 | 1,34 | 2,69 |
| 2004-2008 | 2,91 | 3,62 | 2,23 | 2,48 | 1,48 | 3,06 |
| 2009-2013 | 3,23 | 3,98 | 2,41 | 2,69 | 1,56 | 3,36 |
| 2014-2018 | 3,98 | 4,70 | 2,73 | 3,15 | 1,74 | 3,96 |
| 1999-2018 | **3,17** | **3,88** | **2,35** | **2,62** | **1,53** | **3,27** |

Supplementary table 2. Yearly, 5-yearly and study period crude UTUC incidence rates and age standardized incidence rates adjusted for different standard populations. Due to the different distributions of different age-spans shown in supplementary table 1, the adjusted incidence rates vary greatly.
