# Supplementary figures and images for: Higher than expected and significantly increasing incidence of upper tract urothelial carcinoma. A population based study
Source: World J Urol. 2021 Jan 9;39(9):3385–91. doi: 10.1007/s00345-020-03576-3 (PMC8510951; doi:10.1007/s00345-020-03576-3)

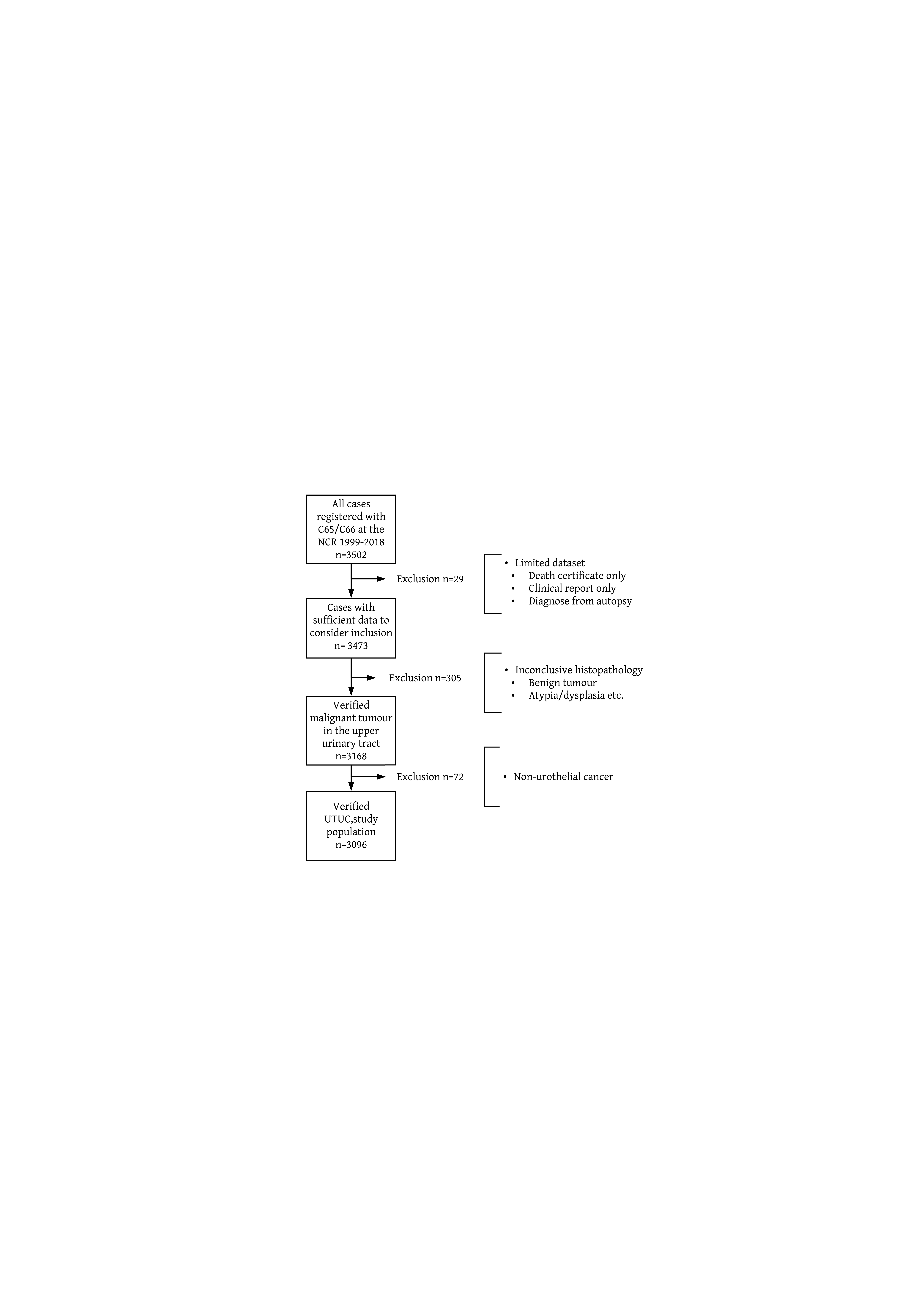

Supplement: Supplementary file 3 — Supplementary file3 (TIF 350 KB) [file 345_2020_3576_MOESM3_ESM.tif]

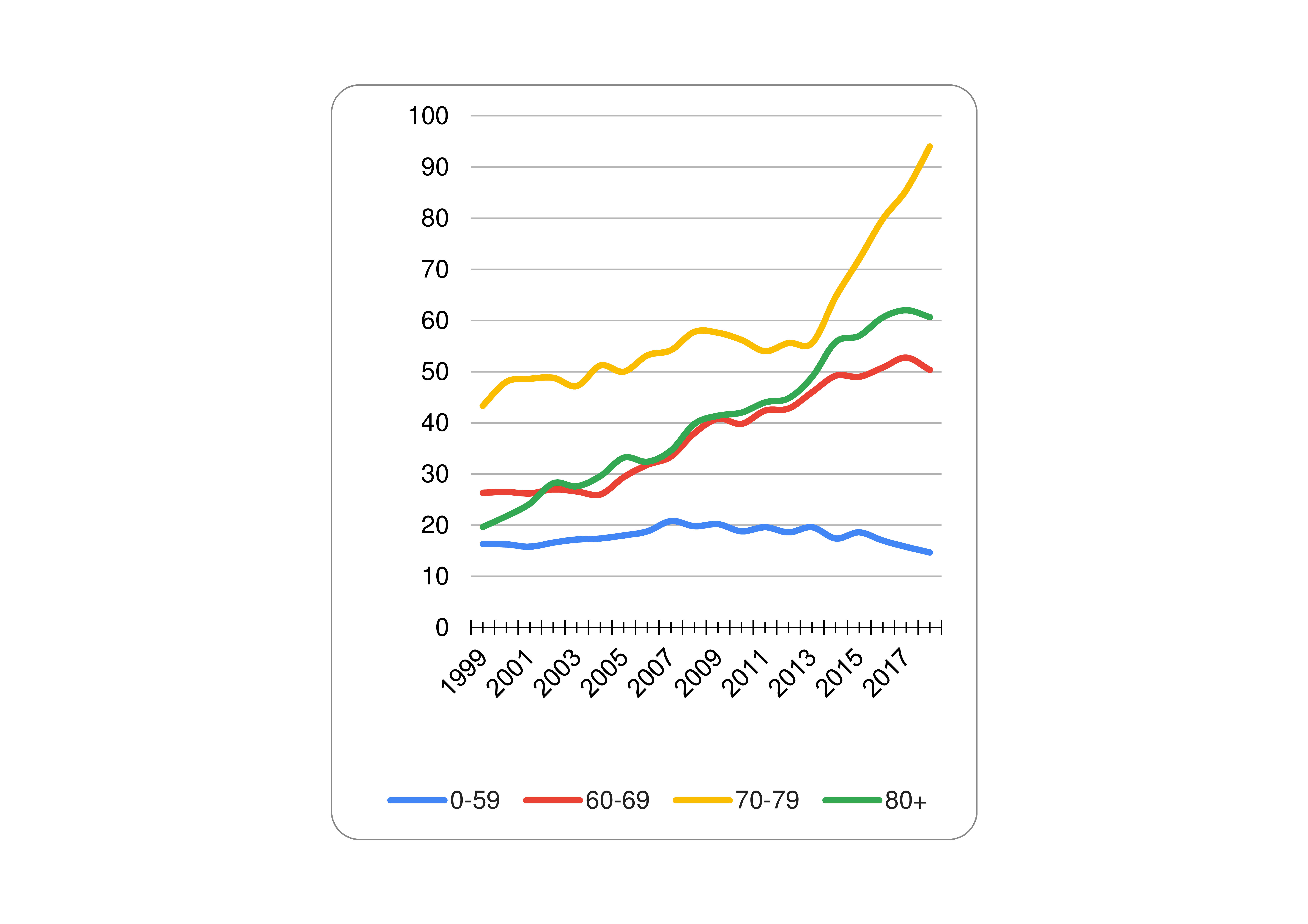

Supplement: Supplementary file 4 — Supplementary file4 (TIF 1495 KB) [file 345_2020_3576_MOESM4_ESM.tif]
